# A novel dual-locus genotyping approach reveals epidemiological characteristics of *Balantioides coli* in intensive pig farms

**DOI:** 10.1016/j.fawpar.2026.e00349

**Published:** 2026-06-11

**Authors:** Suhui Hu, Weifeng Qian, Zhenzhen Liu, Qihao Zhang, Binghui Ding, Chaochao Lv, Min Zhang, Haiyan Wang, Wenchao Yan

**Affiliations:** a College of Animal Science and Technology, Henan University of Science and Technology, Luoyang 471023, China; b Henan University of Animal Husbandry and Economy, 450046, China

**Keywords:** Balantioides coli, Β-Tubulin, ITS, Haplotype, Geographical distribution

## Abstract

*Balantioides coli* is a significant zoonotic parasitic protozoan, with pigs serving as its reservoir host. The limited molecular typing tools for *B. coli* have hindered understanding of its transmission and genetic variability in intensive pig farming. In this study, 1251 pig fecal samples were collected in two batches from three intensive farms. A dual-locus molecular typing method based on the β-tubulin and ITS genes was developed and applied to analyze the epidemiological characteristics and genetic variability of *B. coli*. The results revealed an overall infection rate of 87.7% (1097/1251), with an age-dependent pattern where lower rates were observed in 1–2-month-old piglets, followed by a rapid increase at 3–4 months, and near 100% prevalence by 5–6 months. Phylogenetic analysis based on the β-tubulin gene delineated three haplotypes (I, II, and III), confirming the previously identified ITS sequence variants A and B, and further resolving the novel sequence variant C. The expression of haplotypes and sequence variants showed a marked geographical distribution pattern, with haplotype I and variant A predominantly concentrated in the Zhejiang region, while haplotype III and variant C were detected in pigs and exclusively found in this area. These findings indicate that *B. coli* transmission is highly prevalent under intensive, high-density rearing conditions, and that the distribution of certain sequence variants shows geographical clustering, which may reflect local ecological or husbandry factors. This study provides important dual-locus genotyping insights for elucidating the transmission mechanisms of *B. coli* within intensive farming systems.

## Highlights

• Dual-locus (β-tubulin/ITS) genotyping improves *B. coli* detection and resolution.
• *B. coli* infection rate is 87.7% in pig farms and reaches ∼100% at 5–6 months.
• ITS sequence variant C is detected and the dual-locus method is applicable.
• β-tubulin haplotype III and ITS variant C occur in pigs (both also in guinea pigs).

## Graphical abstract

**Figure:**
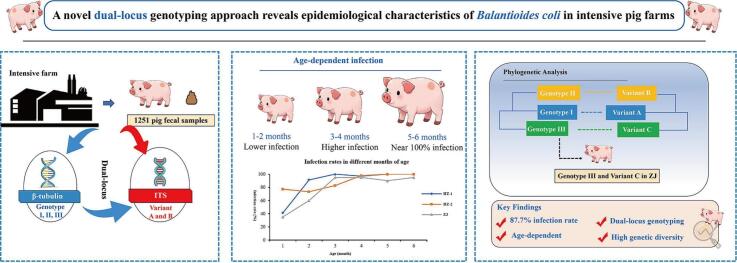

## 1 Introduction

*Balantioides coli* is a neglected zoonotic parasitic protozoan, with a wide range of hosts, mainly pigs and non-human primates (Byun et al., 2021; Ponce-Gordo and Garcia-Rodriguez, 2021). *Balantioides coli* infections have been reported globally in humans and animals, particularly in tropical and subtropical regions (Esteban et al., 1998; Owen, 2005), with prevalence reaching up to 29.0% in humans and 100% in animals (Ahmed et al., 2020). *Balantioides coli* infection in humans primarily manifests as dysentery, though extraintestinal infections involving the peritoneum, genitourinary tract, and lungs may occur (Sharma and Harding, 2003; Kapur et al., 2016; Kaur and Gupta, 2016; Almaw et al., 2022). In swine populations, the parasite generally causes subclinical infections but may act as an opportunistic pathogen, inducing mucosal ulcers, dysentery, and growth retardation, resulting in substantial economic losses to intensive pig farming operations (Allievi et al., 2025).

Currently, research on *B. coli* has not obtained widespread attention, and available data remain scarce. Traditional diagnostic methods (e.g., flotation and sedimentation techniques) exhibit significant limitations, including low sensitivity, an inability to analyze genetic characteristics, and challenges in discriminating morphologically similar pathogens (Ahmed et al., 2020; Li et al., 2020; Ponce-Gordo and Garcia-Rodriguez, 2021). Recently, molecular diagnostic approaches of *B. coli* based on small subunit ribosomal RNA (SSU rRNA) and the internal transcribed spacer (ITS, including the ITS1–5.8S rRNA-ITS2 region) have been developed. The SSU rRNA gene provides a robust marker for species-specific identification, while sequence analysis of the ITS region distinguishes two sequence variants (A and B), (Ponce-Gordo et al., 2008; Ponce-Gordo et al., 2011). However, because the ITS region is non-coding and multicopy, multiple variants may coexist within a single cell, and the predominant variant detected by PCR may not represent the entire genetic content of the isolate. Furthermore, its high mutation rate, while useful for fine-scale genotyping, can complicate phylogenetic inference at deeper evolutionary levels. In previous study, we developed and applied a novel molecular typing tool for *B. coli* based on the β-tubulin gene locus (Hu et al., 2025). Earlier research has demonstrated that β-tubulin exhibits advantages over the conventional ITS marker in terms of typing resolution, genetic polymorphism, and subtype discrimination capability. Therefore, this study innovatively combined β-tubulin with the ITS marker to improve the accuracy and reliability of *B. coli* identification.

So far, at least 11 species have been confirmed as hosts for *B. coli*, with swine being identified as the predominant reservoir (Ponce-Gordo and Garcia-Rodriguez, 2021). Under the increasing intensification of closed confinement farming systems on swine farm, the transmission rate of fecal-oral parasitic diseases is significantly elevated, substantially complicating disease prevention and control efforts. To date, *B. coli* infections have been reported in swine across more than 30 countries worldwide (Ahmed et al., 2020). In this study, we collected fecal samples from pigs aged 1–6 months across three intensive swine farms to investigate the epidemiological patterns and potential risk factors of *B. coli*. We developed a β-tubulin-ITS dual-marker genotyping method to characterize infection prevalence, distribution patterns of sequence variants, and genetic variability across different age groups. The findings provide useful information for risk assessment of *B. coli*.

## 2 Materials and methods

### 2.1 Fecal specimen collection

From October 2024 to March 2025, a total of 1251 fresh fecal samples were collected in two batches from three intensive pig farms (labeled as ZJ, HN-1, and HN-2, representing Zhumadian and Xinyang in Henan province, and Quzhou in Zhejiang province, respectively). The first batch comprised 382 samples systematically collected from pigs aged 1–6 months (∼20 samples per age group), with one sample per pen to ensure independence. The second batch included supplemental sampling (869 samples) at farms HN-1 and HN-2, covering the same age range but allowing multiple samples per pen under a random sampling strategy. For each sample, we recorded the farm of origin, age, and pen number. No direct animal handling or restraint was involved during sample collection. All samples were taken from naturally voided feces immediately after defecation. The study was conducted with the permission of the farm owners. All specimens were immediately transported to the laboratory, preserved in 2.5% potassium dichromate at 4 °C, and stored until DNA extraction.

### 2.2 Genomic DNA extraction and PCR amplification

Genomic DNA was extracted from each fecal sample using the E.Z.N.A.® Stool DNA Kit (Omega Biotek, USA) according to the manufacturer's protocol and stored at −20 °C until further analysis.

The molecular detection of *B. coli* was performed through PCR amplification targeting the ITS region using primers B5D (5’-GCTCCTACCGATACCGGGT-3′) and B5RC (5’-GCGGGTCATCTTACTTGATTTC-3′) (Ponce-Gordo et al., 2011) under the following conditions: each 25 μL reaction contained 12.5 μL of 2× Taq Master Mix, 1 μM each primer, 1 μL DNA template, and 9.5 μL nuclease-free water; thermal cycling: 95 °C for 4 min; 35 cycles of 95 °C for 1 min, 56 °C for 1 min, and 72 °C for 1 min; final extension at 72 °C for 5 min. For the β-tubulin gene, a nested PCR approach was used with external primers F1/R1 (5’-AACTGGGCTAAGGGACACTA-3′/5’-CTCCATTTCGTCCATACCTT-3′) and internal primers F2/R2 (5’-GACCTTCGCTGTCTTCCC-3′/5’-TTCTCCGGTGTACCAATGT-3′). The primary PCR (25 μL) contained 12.5 μL of 2× Taq-PCR-StarMix, 1 μL each of F1/R1, 9.5 μL ddH₂O, and 1 μL DNA template; thermal cycling: 94 °C for 5 min; 35 cycles of 94 °C for 40 s, 53 °C for 40 s, and 72 °C for 1 min; final extension at 72 °C for 10 min. The secondary PCR used the same mix with nested primers F2/R2 and 1 μL of primary PCR product as template, under identical cycling conditions except that the extension time was reduced to 55 s at 72 °C. Sterile water and a confirmed *B. coli*-positive sample served as negative and positive controls, respectively. For the first batch of samples, these controls were used for quality control purposes to verify the functionality of the PCR reaction (e.g., enzyme activity, absence of inhibitors) and to monitor for cross-contamination, thereby avoiding unnecessary sequencing of failed or contaminated reactions. For the second batch, the controls served both to validate the PCR and to serve as diagnostic references.

### 2.3 Sequencing and phylogenetic analysis

The positive PCR amplicons were directly sequenced bidirectionally with Sanger sequencing by GENEWIZ (Suzhou, China). For the first batch of 382 samples, we randomly selected five positive samples per age group (1–6 months) from each farm for both ITS and β-tubulin loci, resulting in 90 samples per marker, and 180 sequences in total. All sequencing chromatograms were visually inspected for double peaks or background noise indicative of mixed sequence variants; no ambiguous chromatograms were observed in the isolates included in the phylogenetic analysis. No sequencing was performed on second-batch samples because the purpose of the second batch was solely intended to investigate prevalence patterns by PCR. The nucleotide sequences of *B. coli* were aligned using Clustal X 2.1 with default parameters. Phylogenetic analysis was conducted in MEGA X by constructing maximum likelihood (ML) trees based on the General Time Reversible (GTR) model, and 1000 bootstrap replicates. Final visualization and annotation of the phylogenetic tree were achieved using the Interactive Tree of Life (iTOL) platform (https://itol.embl.de/).

### 2.4 Statistical analysis

Statistical analyses were performed using SPSS 25.0 (SPSS Inc., Chicago, USA). Pearson's chi-square test (*χ*^*2*^ test) was employed to evaluate prevalence differences among different age groups and geographical regions, with a *P* value <0.05 considered statistically significant.

### 2.5 Nucleotide sequence accession numbers

The β-tubulin gene sequences of *B. coli* obtained from pigs in this study have been deposited in GenBank under accession numbers PX736767 to PX736788, corresponding to isolates 1006, 1172, 998, 1101, 1135, 1182, 1241, 1229, 1064, 1209, 1261, 1130, 1142, 1116, 1262, 1154, 1219, 1040, 1195, 1079, 1215, 1203, respectively. Additionally, the ITS sequences obtained in this study have also been deposited under accession numbers PZ447551 to PZ447557, corresponding to isolates 1195, 1067, 998, 1270, 1135, 1203, 1261, respectively.

## 3 Results

### 3.1 Sequencing results

In this study, 1251 samples were analyzed for *B. coli* using the ITS and β-tubulin genetic loci. *B. coli* was identified in 1097 of the 1251 (87.7%) samples (Table 1). Of these, any sample from the first batch that was positive for only one locus (33 ITS-only, 23 β-tubulin-only) was also sequenced to confirm the result. Thus, a total of 236 sequences were obtained from the first batch. However, only 64 samples yielded successful bidirectional sequences for both loci simultaneously. Not all PCR-positive samples were sequenced. Sequencing was performed only on a subset of samples from the first batch (*n* = 236) to determine sequence variants and haplotypes.

**Table 1:** Overall prevalence and sequence variant/haplotype distribution of *B. coli* in pigs across different regions and age groups.

Area	Age (M)	Sample (n)	Positive (n)	*B. coli* (ITS)			*B. coli* (β-tubulin)			Prevalence (%)
				Prevalence (%)	Positive (n)	Sequence variant (n)	Prevalence (%)	Positive (n)	Haplotype (n)	
HN-1	1	97	40	30.9	30	B (10)	19.6	19	–	41.2
	2	104	95	82.7	86	A (2), B (6)	72.1	75	II (2)	91.3
	3	101	101	100	101	A (1), B (9)	86.1	87	I (3), II (8)	100.0
	4	100	97	94.0	94	–	86.0	86	–	97.0
	5	102	102	99.0	101	B (4)	99.0	101	II (5)	100.0
	6	106	106	90.6	96	B (5)	100.0	106	II (6)	100.0
	total	610	541	82.8	508	A (3), B (34)	78.2	474	I (3), II (21)	88.7
HN-2	1	75	58	61.3	46	B (3)	57.3	43	I (1), II (4)	77.3
	2	90	66	60.0	54	B (5)	41.1	37	II (4)	73.3
	3	92	76	70.7	65	A (3), B (1)	72.8	67	I (4), II (5)	82.6
	4	93	91	93.5	87	A (1), B (5)	96.8	90	I (1), II (4)	97.8
	5	95	95	96.8	92	B (5)	88.4	84	II (5)	100.0
	6	76	76	94.7	72	B (5)	93.4	71	II (5)	100.0
	total	521	462	80.2	416	A (4), B (24)	75.2	392	I (6), II (27)	88.7
ZJ	1	20	7	20.0	4	B (1), C (2)	30.0	6	II (2)、III (3)	35.0
	2	20	12	50.0	10	B (5)	55.0	11	II (6)	60.0
	3	20	19	70.0	14	A (2), B (3)	95.0	19	I (3), II (7)	95.0
	4	20	19	95.0	19	A (1), B (7)	80.0	16	I (1), II (6)	95.0
	5	20	18	85.0	17	A (9), B (1)	90.0	18	I (11), II (1)	90.0
	6	20	19	95.0	19	–	95.0	19	–	95.0
	total	120	94	70.8	83	A (12), B (17), C (2)	74.2	89	I (15), II (22), III (3)	78.3
Total		1251	1097	80.5	1007	A (19), B (75), C (2)	76.3	955	I (24), II (70), III (3)	87.7

Two genetic loci were used for sequence variant identification and genotyping. The β-tubulin locus detected 955 positive samples (76.3%), while the ITS locus detected 1007 positive samples (80.5%). Three haplotypes were identified based on the β-tubulin locus, including haplotype I (*n* = 24), haplotype II (*n* = 70) and haplotype III (*n* = 3). Haplotype II was the dominant haplotype across all age groups, while haplotype I was only detected in the ZJ region. The ITS locus analysis showed that sequence variant B (*n* = 75) was the dominant variant, while sequence variant A (*n* = 19) was sporadically distributed and sequence variant C (n = 2) also only identified in ZJ. (Table 1).

The highest infection rate was 100%, identified in 3-, 5- and 6-month-old pigs in the HN-1 and in 5- and 6-month-old pigs in the HN-2. *B. coli* infection was significantly correlated with age (χ^2^ = 24.296, *p* < 0.001). The infection rates in HN-1 (541/610; 88.7%) and HN-2 (462/521; 88.7%) were higher than that in ZJ (94/120; 78.3%). A highly significant difference in *B. coli* infection prevalence was observed among the farms (χ^2^ = 2502.00, *p* < 0.001).

### 3.2 *Balantioides coli* occurrence identified by first batch survey

A total of 382 samples were collected to characterize the infection dynamics of *B. coli*, with an overall infection rate of 83.2%. Moreover, in the first batch of samples, 56 isolates were positive for only one of the two markers (33 for ITS and 23 for β-tubulin), all of which were confirmed as *B. coli* by sequencing.

A consistent age-related infection pattern was observed across HN-1 and ZJ regions. Infection rates were low in 1- to 2-month-old piglets, increased rapidly in 3- to 4-month-old pigs, and remained consistently high in 5- to 6-month-old pigs. The lowest infection rate was recorded at 1 month of age, with HN-1 at 45.8%, and ZJ at 35.0%. Infection rates rose rapidly between 2 and 3 months, reaching 100% in HN-1 by 3 months. From 4 to 6 months, all regions maintained high infection levels, with rates exceeding 90% in most age groups (Table 2).

**Table 2:** Prevalence and sequence variant/haplotype distribution of *B. coli* in pigs across different regions and age groups (First batch).

Area	Age (M)	Sample (n)	Positive (n)	*B. coli* (ITS)			*B. coli* (β-tubulin)			Prevalence (%)
				Prevalence (%)	Positive (n)	Sequence variant (n)	Prevalence (%)	Positive (n)	Haplotype (n)	
HN-1	1	24	11	45.8	11	B (10)	4.17	1	–	45.8
	2	25	22	80.0	20	A (2), B (6)	48.0	12	II (2)	88.0
	3	22	22	100	22	A (1), B (9)	100.0	22	I (3), II (8)	100.0
	4	20	20	90.0	18	–	100.0	20	–	100.0
	5	21	21	100	21	B (4)	100.0	21	II (5)	100.0
	6	21	21	100	21	B (5)	100.0	21	II (6)	100.0
HN-2	1	15	12	60.0	9	B (3)	73.3	11	I (1), II (4)	80.0
	2	21	16	71.4	15	B (5)	61.9	13	II (4)	76.2
	3	24	12	33.3	8	A (3), B (1)	41.7	10	I (4), II (5)	50.0
	4	24	22	91.7	22	A (1), B (5)	87.5	21	I (1), II (4)	91.7
	5	25	25	100	25	B (5)	100.0	25	II (5)	100.0
	6	20	20	100	20	B (5)	95.0	19	II (5)	100.0
ZJ	1	20	7	20.0	4	B (1), C (2)	30.0	6	II (2)、III (3)	35.0
	2	20	12	50.0	10	B (5)	55.0	11	II (6)	60.0
	3	20	19	70.0	14	A (2), B (3)	95.0	19	I (3), II (7)	95.0
	4	20	19	95.0	19	A (1), B (7)	80.0	16	I (1), II (6)	95.0
	5	20	18	85.0	17	A (9), B (1)	90.0	18	I (11), II (1)	90.0
	6	20	19	95.0	19	–	95.0	19	–	95.0
Total		382	318	77.2	295	A (19), B (75), C (2)	74.6	285	I (24), II (70), III (3)	83.2

Regional dynamics revealed distinct patterns. HN-1 exhibited early and sustained high infection, starting at 45.8% at 1-month-old, increasing to 100% by 3-month-old, and maintaining this level thereafter. HN-2 displayed a fluctuating early pattern, with rates ranging from 50.0% to 91.7% from 1 to 4 months, before stabilizing at 100% by 5–6 months. ZJ showed a steady incremental trend, rising consistently from 35.0% at 1 month to 95.0% at 6 months. Both molecular detection methods, the ITS and β-tubulin loci, yielded highly concordant results across regions. Each method detected sustained 100% prevalence in HN-1 from three months of age and near-complete detection in HN-2 from five months of age. In ZJ, both methods demonstrated a gradual monthly increase in detection rates (Table 2).

At the ITS locus, Sequence variant B was predominant across all age groups in all three regions. Sequence variant A showed a sporadic distribution with localized clustering in 5-month-old pigs in the ZJ region (9/19), whereas Sequence variant C was exclusively identified in 1-month-old pigs. At the β-tubulin locus, haplotype II was predominant across all age groups in both HN-1 and HN-2 regions, whereas haplotype I primarily detected in the ZJ region (15/24). The rare haplotype III was exclusively found in the ZJ region (3/3) (Table 2).

### 3.3 *Balantioides coli* occurrence identified by second batch survey

A second sampling was conducted to further investigate *B. coli* infection in the HN-1 and HN-2 regions. A total of 869 samples were collected, with an overall infection rate of 89.6% (779/869). The infection rates were 88.9% (424/477) in HN-1 and 90.6% (355/392) in HN-2 (Table 3).

**Table 3:** Prevalence of *B. coli* in pigs across different regions and age groups (second batch).

Area	Age (M)	Sample (n)	Positive (n)	*B. coli* (ITS)		*B. coli* (β-tubulin)		Prevalence (%)
				Prevalence (%)	Positive (n)	Prevalence (%)	Positive (n)	
HN-1	1	73	29	26.0	19	24.7	18	39.7
	2	79	73	83.5	66	79.7	63	92.4
	3	79	79	100	79	82.3	65	100.0
	4	80	77	95.0	76	82.5	66	96.3
	5	81	81	98.8	80	98.8	80	100.0
	6	85	85	88.2	75	100	85	100.0
HZ-2	1	60	46	61.7	37	53.3	32	76.7
	2	69	50	56.5	39	34.8	24	72.5
	3	68	64	83.8	57	83.8	57	94.1
	4	69	69	94.2	65	100	69	100.0
	5	70	70	95.7	67	84.3	59	100.0
	6	56	56	92.9	52	92.9	52	100.0
Total		869	779	81.9	712	77.1	670	89.6

The lowest rates were observed at one month of age with 39.7% in HN-1 and 76.7% in HN-2. From two months, infection rates rose rapidly, reaching nearly or fully 100.0% in both regions by 5–6 months of age. Based on detection using the ITS and β-tubulin markers, the overall positivity rates were 81.9% (712/869) for ITS and 77.1% (670/869) for β-tubulin. In HN-1, at 3 months of age, the positivity rate was 100.0% by ITS but only 82.3% by β-tubulin (Table 3).

### 3.4 Phylogenetic relationship of *Balantioides coli* isolates

**Fig. 1:**
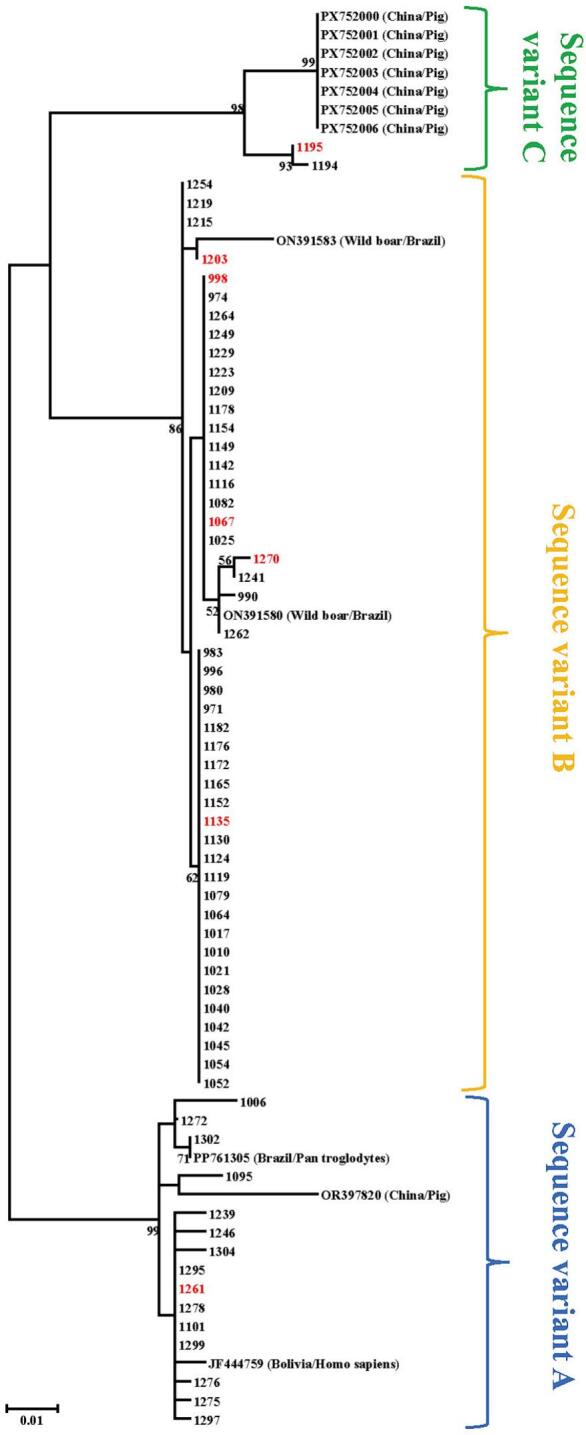
Phylogenetic tree constructed based on ITS sequences. The tree was constructed under the General Time Reversible (GTR) nucleotide substitution model. Bootstrap support values (1000 replicates) are shown at the nodes (values <50% are not displayed). Colors indicate ITS sequence variants: blue for variant A, yellow for variant B, and green for variant C. All sequences labeled with numbers in the tree were obtained from this study. Numbers shown in red correspond to representative sequences of each group deposited in GenBank. (For interpretation of the references to colour in this figure legend, the reader is referred to the web version of this article.)

To enable a direct and meaningful comparison of phylogenetic relationships between the two markers, we restricted the phylogenetic analysis to these 64 samples that had complete paired sequence data for both ITS and β-tubulin markers. In the ITS-based phylogenetic tree, all sequences were primarily divided into three clades: sequence variant A, B and C. Variant A clustered with a reference sequence (PP761305, OR397820, JF444759), while variant B, together with another reference sequence (ON391580, ON391583), formed a well-supported independent branch. Sequence variant C, although comprising only two isolates (1194 and 1195), also formed an independent clade with high bootstrap support (98.0%) (Fig. 1).

**Fig. 2:**
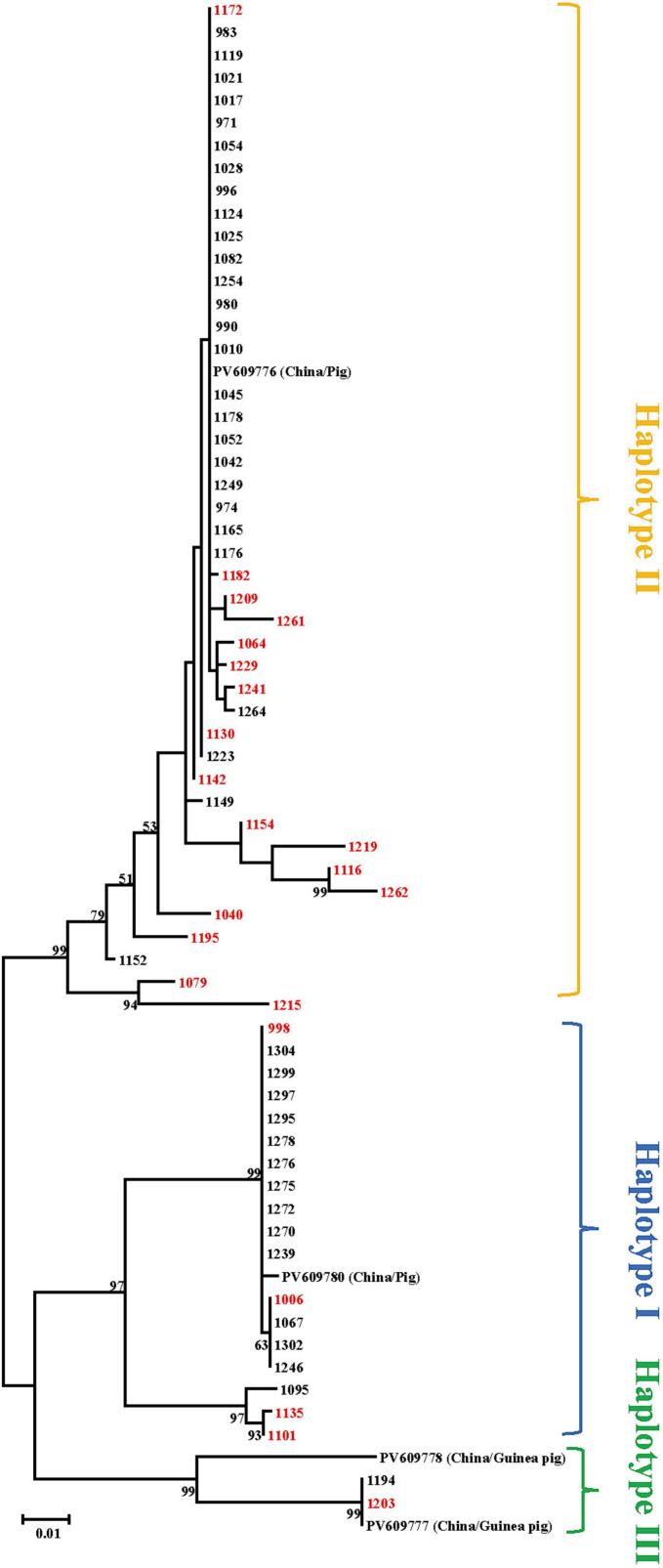
Phylogenetic tree constructed based on β-tubulin sequences. The tree was constructed under the General Time Reversible (GTR) nucleotide substitution model. Bootstrap support values (1000 replicates) are shown at the nodes (values <50% are not displayed). Colors indicate β-tubulin haplotypes: blue for haplotype I, yellow for haplotype II, and green for haplotype III. All sequences labeled with numbers in the tree were obtained from this study. Numbers shown in red correspond to representative sequences of each group deposited in GenBank. (For interpretation of the references to colour in this figure legend, the reader is referred to the web version of this article.)

Phylogenetic analysis based on the β-tubulin locus classified the isolates into three well-supported haplotypes (I, II, and III). Analysis of the ITS locus distinguished three variants (A, B, and C); variants A and B have been previously recognized, and variant C (previously reported in guinea pigs) was also found in pigs. Haplotype I showed close clustering with the reference sequence PV609780. In contrast, haplotype II and haplotype III each formed distinct branches, with haplotype II containing the reference sequence PV609776, and haplotype III encompassing the reference sequences PV609778 and PV609777 (Fig. 2).

## 4 Discussion

This study investigated the prevalence of *B. coli* in three intensive pig farms in China using a novel dual-locus method that combines a newly established β-tubulin genotyping assay with conventional ITS typing. The research revealed an exceptionally high infection rate and an age-dependent infection pattern on these intensive farms. Notably, phylogenetic analysis based on the β-tubulin locus resolved the isolates into three distinct haplotypes (I, II, and III), demonstrating the utility of this protein-coding marker for genotyping of *B. coli*. In addition, we detected haplotype III in porcine-derived *B. coli* for the first time. To our knowledge, this haplotype had previously been reported only in guinea pigs (Hu et al., 2025). This finding suggests that this β-tubulin sequence variant is not strictly host-specific.

The overall prevalence of *B. coli* in this study was 87.7%. This rate is substantially higher than the previously reported in Brazil (27.5%, 325/1177) (Barbosa Ada et al., 2015), Korea (64.7%, 88/136) (Ismail et al., 2010), Bangladesh (38.3%, 62/162) (Dey et al., 2014), Ghana (19.3%, 50/259) (Permin et al., 1999), Nigeria (51.5%, 207/402) (Yatswako et al., 2007), Turkey (1.6%, 4/238) (Uysal et al., 2009), Germany (0.7%, 2/287) (Wieler et al., 2001) and China (26.5%, 5187/19593) (Zhang et al., 2023) but lower than rates reported in India (93.0%, 93/100) (Bauri et al., 2012) and Italy (92.4%, 813/880) (Allievi et al., 2025). This suggests that under the global epidemic background of African Swine Fever, intensive, high-density, closed farming systems have greatly facilitated the spread of *B. coli*. Haplotype.

This study revealed an age-dependent distribution pattern of *B. coli* infection. The infection rate was relatively low in 1–2 month-old piglets, increased rapidly in 3–4 month-old pigs, and remained close to 100.0% in 5–6 month-old pigs. This pattern was consistently validated across both the first (*n* = 382) (except in HN-2) and second (*n* = 869) sample batches. These findings are consistent with reports from Shaanxi, Hunan, and Guangdong provinces in China, where the highest infection rates were detected in fattening pigs (Li et al., 2020). Some studies have reported the highest prevalence in sows from Henan, Guangdong, and Jiangxi (Weng et al., 2005; Qiu et al., 2008; Zhang et al., 2018). Others found the highest rates in suckling piglets in Henan (Wu et al., 2007) and post-weaning piglets in Sichuan (Lai et al., 2011). These variations likely result from a complex interplay of factors, including herd immunity status, lactation management, weaning protocols, and pen hygiene across production sites.

The dual-locus detection strategy combining ITS and β-tubulin employed in this study significantly enhanced diagnostic accuracy and the ability to resolve genetic variability. The overall infection rate (87.7%) was calculated based on the principle that positivity at either locus indicates infection. The individual detection rates for ITS and β-tubulin were 80.5% and 76.3%, respectively. Moreover, in the first batch of samples, 56 isolates were positive for only one of the two markers (33 for ITS and 23 for β-tubulin), all of which were confirmed as *B. coli* by sequencing. This indicates that using a single marker may lead to an underestimation of prevalence.

In the present study, the detection rate for the ITS locus (80.5%) was higher than β-tubulin (76.3%). This discrepancy is likely due to the multicopy nature of the rRNA gene cluster, which increases PCR sensitivity. The dual-locus strategy primarily enhances detection sensitivity and reduces the risk of underestimation that may occur when relying on a single marker, as positivity at either locus was considered indicative of infection.

Analysis of the 64 isolates revealed that 57 conformed to the pattern in which sequence variant A correlated with haplotype I and variant B with haplotype II. Notably, the present study identified two isolates belonging to variant C, a phylogenetically distinct sequence variant previously reported in guinea pigs (Hu et al., 2025). This finding supports the distinct existence of variant C and suggests a possible association with β-tubulin haplotype III, although this association is based on only two samples. In present study, seven isolates exhibited discordant typing. This deviation may stem from variation in evolutionary properties across genetic loci. Specifically, the protein-coding β-tubulin gene and the non-coding ITS region differ in their evolutionary rates and selective pressures, which can lead to discordant phylogenetic signals (Kellis et al., 2014). Furthermore, genetic recombination may cause the evolutionary history of a single gene to fail to recover the organism's full phylogeny (Lynch and Conery, 2003).

This study revealed a marked geographical distribution pattern in the haplotype distribution of *B. coli* in pigs. Haplotype I was predominantly concentrated in the ZJ region (15/24), while haplotype III was exclusively detected in this region (3/3). Meanwhile, the frequency of ITS variant A was also highest in the ZJ region (12/19) and variant C was only identified in ZJ region (2/2). This clustered distribution pattern suggests that geographical isolation or locally distinct farming ecosystems may have shaped a unique population structure of *B. coli* in this area. Notably, haplotype III and variant C were detected for the first time in pigs during this study, potentially representing a recently emerged local haplotype or a lineage introduced via cross-species transmission from other animal hosts such as guinea pigs (Hu et al., 2025). These findings highlight the spatial association of specific genotypes with defined geographical regions and suggest that further investigation into spatial patterns may be informative for local surveillance efforts.

## 5 Conclusions

This study investigated *B. coli* infection in intensive pig farms in China using a novel dual-locus genotyping method targeting the β-tubulin and ITS genes. Analysis of 1251 fecal samples revealed a high overall infection rate of 87.7%, with prevalence increasing with age and reaching near 100% in pigs aged 5–6 months. Genetic sequencing identified three β-tubulin haplotypes (I, II, III) and detected ITS sequence variants A, B, and C (variant C previously reported in guinea pigs). The distribution of haplotypes and sequence variants exhibited a geographical pattern, with haplotype I/variant A predominant and haplotype III/variant C detected only in the Zhejiang region. These findings demonstrate that *B. coli* transmission is widespread under intensive farming conditions and that the distribution of certain sequence variants shows geographical clustering, which may reflect local ecological or husbandry factors.

## CRediT authorship contribution statement

**Suhui Hu:** Writing – review & editing, Writing – original draft, Funding acquisition, Conceptualization. **Weifeng Qian:** Writing – review & editing, Funding acquisition. **Zhenzhen Liu:** Writing – original draft, Methodology, Data curation. **Qihao Zhang:** Data curation. **Binghui Ding:** Data curation. **Chaochao Lv:** Writing – review & editing. **Min Zhang:** Writing – review & editing. **Haiyan Wang:** Writing – review & editing, Formal analysis, Conceptualization. **Wenchao Yan:** Writing – review & editing, Supervision, Conceptualization.

## Declaration of competing interest

All authors declare that they have no conflicts of interest regarding the publication of this manuscript. No financial or personal relationships with other people or organizations have inappropriately influenced this work.

## Data Availability

All relevant data supporting our findings are provided in the main text files. The newly determined β-tubulin and ITS sequences have been deposited in the NCBI database under accession numbers PX736767 to PX736788, and PZ447551 to PZ447557.
